# ECOLOGICAL MOMENTARY ASSESSMENT OF SOCIAL INTERACTION, LONELINESS, AND FRAILTY IN PREDEMENTIA

**DOI:** 10.1093/geroni/igae098.2756

**Published:** 2024-12-31

**Authors:** Dahye Hong, Jennifer Kim, Seolah Yoon, Chaeeun Kang, Bada Kang

**Affiliations:** Yonsei University, Seoul, Republic of Korea; Yonsei University, Seoul, Republic of Korea; Yonsei University, Seoul, Republic of Korea; Yonsei University, Seoul, Republic of Korea; Yonsei University, Seoul, Republic of Korea

## Abstract

The pre-dementia stage, encompassing subjective cognitive decline (SCD) and mild cognitive impairment (MCI), is recognized as being vulnerable to frailty and is associated with adverse health outcomes, such as falls or disability. Although social interaction and loneliness are considered to be associated with frailty, little is known about the relationship between social interaction, loneliness, and frailty in the pre-dementia stage. Therefore, this study examined the relationship between social interaction, loneliness, and frailty among 101 community-dwelling older adults in the pre-dementia stage (individuals with SCD = 67; individuals with MCI = 34) in Korea. To capture real-time measurements of social interaction and loneliness, we utilized an ecological momentary assessment that involved repeated sampling of individuals’ states. Participants reported their frequency of social interactions and loneliness via a mobile application four times a day for two weeks. Multivariate logistic regression analysis was used to investigate the associations between social interaction, loneliness, and frailty. We adjusted for mild behavioral impairment (MBI), which indicates dementia risk and increases vulnerability to frailty, alongside sociodemographic, health-related characteristics, and functional limitations. Our study demonstrated that social interaction has a negative association with frailty among individuals in the pre-dementia stage, regardless of the presence or severity of MBI symptoms. However, loneliness was not significantly associated with frailty. This study highlights that the assessment of social interactions using ecological assessment will help identify at-risk individuals and implement targeted interventions for the prevention and management of frailty in older adults in the pre-dementia stage.

